# Integration of Rehabilitation Activities Into Everyday Life Through Telerehabilitation: Qualitative Study of Cardiac Patients and Their Partners

**DOI:** 10.2196/13281

**Published:** 2019-04-15

**Authors:** Birthe Dinesen, Gitte Nielsen, Jan Jesper Andreasen, Helle Spindler

**Affiliations:** 1 Laboratory of Welfare Technologies - Telehealth and Telerehabilitation Department of Health Science and Technology Aalborg University Aalborg Denmark; 2 Department of Cardiology Region Hospital North Jutland Hjoerring Denmark; 3 Department of Cardiothoracic Surgery Aalborg University Hospital Aalborg Denmark; 4 Department of Clinical Medicine Aalborg University Hospital Aalborg Denmark; 5 Department of Psychology and Behavioral Sciences Aarhus University Aarhus Denmark

**Keywords:** telerehabilitation, heart diseases, social media, qualitative study

## Abstract

**Background:**

Implementation of cardiac rehabilitation has not been optimal, with patient participation rates below 50%. Factors that contribute to cardiac patients’ lack of participation in rehabilitation programs are patient motivation, logistical difficulties in getting to the rehabilitation facilities, lack of psychosocial elements, and individualization of activities in the rehabilitation programs. Telerehabilitation has been proposed as a new way to address the challenge of engaging and motivating cardiac patients and their partners to participate in rehabilitation.

**Objective:**

The aim of this study was to explore the experiences of cardiac patients and their partners of participating in the Teledialog Telerehabilitation Program (TTP). The Teledialog program consisted of a digital rehabilitation plan, transmission of health data from patient’s home to hospital and health care center, and an interactive Web portal with information and training videos.

**Methods:**

This case study used a theoretical approach combining the “community of practice” approach and self-determination theory. A triangulation of data collection techniques was used, including documents, participant observation (72 hours), and qualitative interviews with cardiac patients and their partners enrolled in the telerehabilitation group. A total of 14 cardiac patients, 12 patient spouses/partners, and 1 son participated in the study. The participants were interviewed at enrollment in the telerehabilitation program and after 12 weeks of participation in the program. Interview data were analyzed using NVivo 11.0.

**Results:**

Patients and their partners found the Web portal ActiveHeart.dk and the electronic rehabilitation (e-rehabilitation) plan to be helpful tools for health education, coordinating rehabilitation goals, creating an overview of the data, and ensuring continuity in the rehabilitation process. The patients felt that the TTP treated them as individuals, gave them a sense of autonomy, and provided enhanced relatedness to health care professionals and partners and a sense of competence as active participants in their own rehabilitation process. Some patients missed being part of a community of practice with other cardiac patients and did not use the Web forum. Patients’ partners found that the telerehabilitation program gave them a sense of security and helped them balance their involvement as a partner to the patient and not push the patient too hard.

**Conclusions:**

Cardiac patients and their partners found telerehabilitation technologies a useful digital toolbox in the rehabilitation process. Telerehabilitation motivated the patients to integrate rehabilitation activities into their work schedule and everyday life and made them feel like unique individuals. Participating in the Teledialog Telerehabilitation Program might not be a suitable strategy for all cardiac patients. Being a patient’s partner in the telerehabilitation program was associated with a heightened sense of security, navigation between active involvement in the rehabilitation process, being an equal partner, and not pushing the patient too hard.

## Introduction

Each year, 17.9 million people die from cardiovascular diseases, and it is estimated that cardiovascular diseases cause 31% of all deaths worldwide [1]. Cardiac rehabilitation programs include interventions such as exercise and patient education on risk factors [2-3]. Cardiac rehabilitation helps patients alter their lifestyle and decrease their mortality rate [2-4]. However, despite international recommendations, effective implementation of cardiac rehabilitation after cardiovascular diseases has not been optimal, with participation rates below 50% [1-3]. Many factors contribute to patients’ lack of participation in rehabilitation activities including patients’ motivation, lack of means of transport to the clinic, time constraints, scheduling commitments associated with returning to work, lack of psychosocial counseling in the programs, lack of individualization of activities in the rehabilitation programs, and lack of involvement of partners [3,5-7]. More innovative models for cardiac rehabilitation are needed to address the challenge of engaging and motivating cardiac patients and their partners to participate in rehabilitation. Telerehabilitation can be one such new approach. Telerehabilitation is defined as the delivery of rehabilitation services via information and communication technologies [8].

A review of internet-based cardiovascular rehabilitation from 2013 [9,10], including a small number of trials and few outcome measures (physical activity, clinical, and psychosocial outcomes), reported positive results with regard to patient outcome and feedback. However, none of the studies were integrated with clinical practice, as confirmed by another review from 2013 [11].

A 2015 review [7] on telerehabilitation among cardiac patients found that several more studies had been carried out, but these studies were characterized as heterogeneous with respect to interventions, patients, and outcome measures. Most interventions had only one or two components of cardiac rehabilitation, and physical activities were the most frequently used approach [7]. Systematic reviews on cardiac telerehabilitation show that it can help reduce depression and improve both functional capacity and physical activity level [12,13].

There is a lack of qualitative studies exploring cardiac rehabilitation participants’ and their partners’ experiences in telerehabilitation programs. Partners of cardiac patients play an important role in patients’ adjustment to lifestyle changes and the rehabilitation process [14,15]. Qualitative knowledge about patients’ and their partners’ experiences of telerehabilitation can be a valuable source for optimizing future cardiac telerehabilitation programs.

From 2011 to 2012, the Danish Teledialog Telerehabilitation Program (TTP) for cardiac patients and their partners was developed through user-driven innovation [16]. The TTP emphasized collaboration between cardiac patients, their partners/relatives, health care professionals, researchers, and representatives from companies. The TTP was developed with inspiration from learning theory, specifically the “communities of practices” approach [17]. The goal was to facilitate mutual learning between patients, partners, and health care professionals in the telerehabilitation program. Self-determination theory (SDT) [18,19] was also applied, with the purpose of exploring whether telerehabilitation could help engage and motivate patients and partners in the rehabilitation process.

The aim of this study was to explore the experiences of cardiac patients and their partners who participated in the TTP.

## Methods

### Design

This study is a substudy of the TTP and was performed as a descriptive case study [20]. According to Yin (2013), a case study is defined as “an empirical inquiry that investigates a contemporary phenomenon (the ‘case’) in depth and within its real-world context, especially when the boundaries between phenomenon and context may not be clearly evident” [20].

### Description of Sampling

The inclusion criteria for enrolling cardiac patients in the Teledialog project were age over 18 years; living in North Jutland in Denmark; history of acute coronary syndrome, heart failure, or coronary artery bypass surgery/valve surgery; living in an area with mobile coverage; and user-level competence in information and communication technologies. The exclusion criteria were inability to speak Danish, pregnancy, or breastfeeding. From May 2013 to January 2014, patients enrolled in the telerehabilitation group of the Teledialog trial were consecutively contacted and invited for interviews. A total of 14 cardiac patients (9 men and 5 women) participated in two interviews: One, at enrollment into the TTP and another, after 12 weeks of participation in the program. A total of 12 patient partners and one son of a patient participated in the interviews. Table 1 shows the baseline characteristics of the interviewed patients and partners.

**Table 1:** Basic characteristics of the interviewed patients and partners from the telerehabilitation group.

Variables		Interviewed patients in the telerehabilitation group (n=14)	Interviewed partners (n=13)
**Age (years), mean (SD); range**			
	Men^a^	61.56 (11.39); 47-85	54 (5.06); 45-60
	Women^b^	58.40 (10.62); 51-77	58,25 (12.27); 40-82
	Total	60.43 (10.82); 47-85	56,62 (10.33); 40-82
Weight (kg)		90.03 (23.706); 43.90-130.00	N/A^c^
**Blood pressure, mean (SD); range**			N/A
	Systolic blood pressure	125.93 (17.76); 90-95	N/A
	Diastolic blood pressure	73.71 (11.22), 58-90	N/A
Heart rate (beats/minute), mean (SD), range		70.143 (7.02), 57-81	N/A
**Condition, n (%)**			
	Acute coronary syndrome	7 (50)	N/A
	Cardiac surgery	4 (28.57)	N/A
	Heart failure	3 (21.43)	N/A
	Arterial sclerosis and heart failure	0 (0)	N/A
**Status, n (%)**			
	Single	1 (7.14)	1^d^ (7.69)
	Married or living with a partner	13 (92.85)	12 (92.30)
**Education, n (%)**			
	Elementary school	2 (14.28)	3 (23.07)
	High school	3 (21.42)	2 (15.38)
	Skilled work	8 (57.14)	6 (46.15)
	Higher education	0 (0)	1 (7.69)
	Bachelor’s degree	0 (0)	0 (0)
	Master’s degree	1 (7.14)	1 (7.69)
**Employment, n (%)**			
	<20 h/week	0 (0)	0 (0)
	20-36 h/week	0 (0)	0 (0)
	37 h/week	4 (28.54)	7 (53.84)
	On sick leave	5 (35.71)	0 (0)
	Unemployed	0 (0.00)	1(7.69)
	Retired	5 (35.71)	5 (38.46)
	Missing data	0 (0)	0 (0)
**Participating institutions, n (%)**			
	Hospital	6 (42.85)	N/A
	Health care center	7 (50.00)	N/A
	Call center	1 (7.14)	N/A

^a^n=9 (patients); n=5 (partners).

^b^n=5 (patients); n=8 (partners).

^c^N/A: not applicable.

^d^Son was interviewed.

Prior to the initial interview, the patients were asked to invite a partner to participate in the interviews. One patient who was single invited a son to participate, as the son was an important part of the patient’s life after cardiac disease.

None of the patients refused to participate in the two interviews or dropped out during the intervention period.

Three patients within the total intervention group (n=75) dropped out during the 12-week telerehabilitation program due to disconfirmation of diagnosis (n=1), inability to cope with the project (n=1), and serious illness (n=1).

### Presentation of the Teledialog Telerehabilitation Program

The TTP followed the current guidelines for cardiac rehabilitation developed by the Danish Health Agency [21,22] and the European Association of Cardiovascular Prevention and Rehabilitation [23]. The overall aims of the TTP were to create more personalized rehabilitation; engage and motivate more cardiac patients to participate in rehabilitation; increase patients’ quality of life; and facilitate coherence between patients, partners, and health care professionals in the rehabilitation process.

Textbox 1 provides an overview of the content (equipment, exercise, and education) of the TTP, which lasted 12 weeks for each patient.

In the TTP, the participating institutions were a cardiology ward at a regional hospital, a thoracic ward at a university hospital, a call center, and four health care centers in two municipalities that participated.

**Figure 1:**
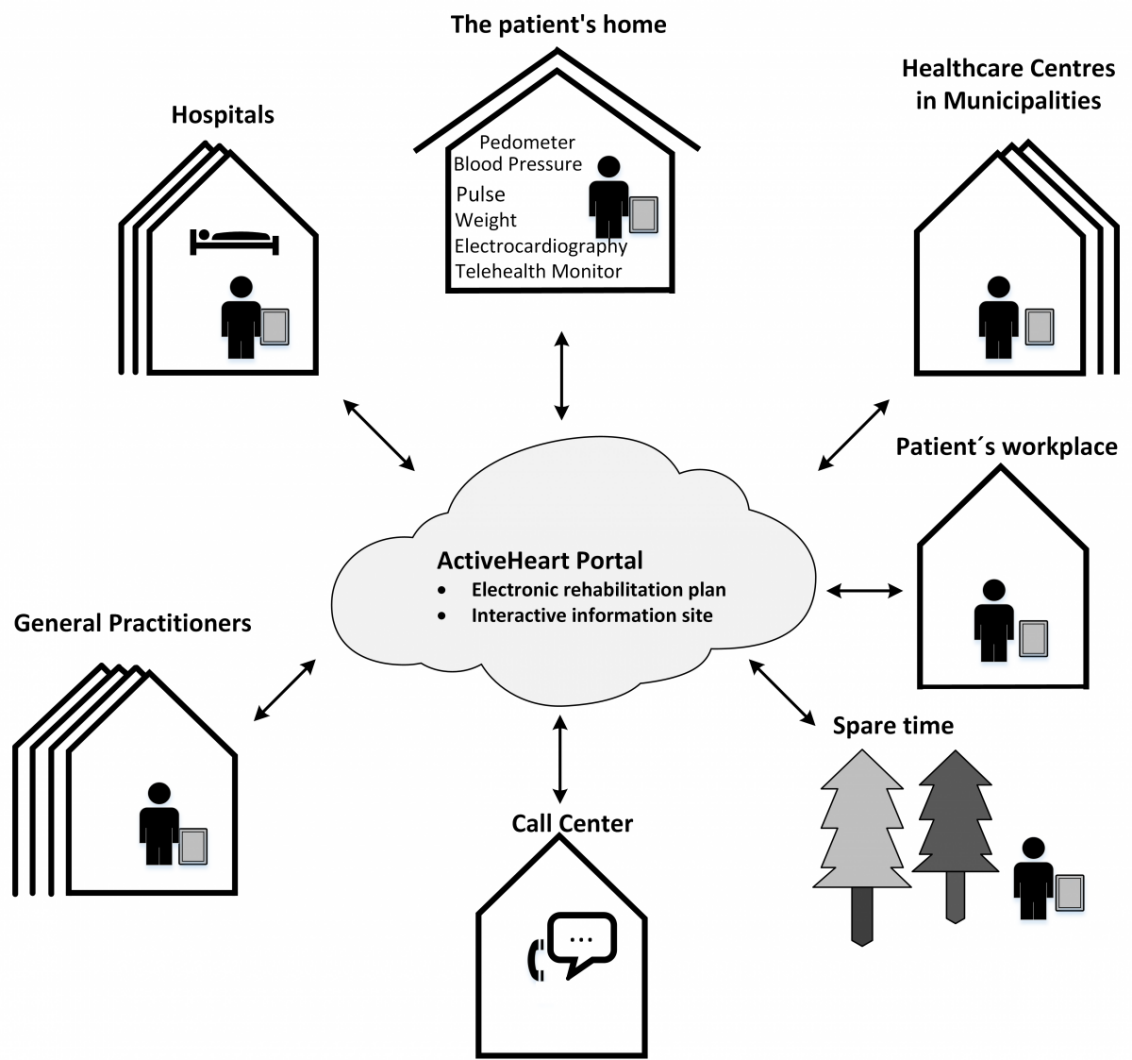
Overview of the Teledialog Telerehabilitation Context.

At discharge from the hospital, the patients and their partners were invited to a meeting with a project nurse. At the meeting, the patient was interviewed to identify their specific needs for rehabilitation. An individualized rehabilitation plan was designed in collaboration with the patients and their partners. Patients could participate in telerehabilitation from hospital, health care center, or call center. They were also allowed to participate in rehabilitation activities at the health care center, for example, as group-based training. They received education on using the devices for measuring blood pressure, pulse, weight, and steps and were taught to navigate the ActiveHeart Web portal, which was the center of the telerehabilitation technologies (Textbox 1, Figure 1). The ActiveHeart is an interactive information site with text and videos on rehabilitation issues. The portal also has a Web forum that enables patients and their partners to communicate with each other. The function of the e-rehabilitation plan is described in Textbox 1. Patients, partners, nurses, doctors from the hospital, physiotherapists, and nurses from the health care center had access to the e-rehabilitation plan. The patient had to give written consent to allow the parties access to the e-rehabilitation plan. During the 12 weeks of rehabilitation, the parties used the e-rehabilitation plan for communication, sharing data on home measurements, information, and goal setting for rehabilitation activities. Each patient had a contact person who was responsible for the collaboration with the individual patient and his/her rehabilitation process.

A video of the TTP is provided in Multimedia Appendix 1. The TTP was tested from 2012 to 2014 in a randomized controlled trial.

**The Teledialog Telerehabilitation Program content.**

Equipment:

- Telehealth monitor approved for medical use and paired with a telehealth monitor in advance
- Sphygmomanometer approved for medical use and paired with a telehealth monitor in advance
- Weight scale approved for medical use and paired with a telehealth monitor in advance
- Device for measuring electrocardiography approved for medical use and paired with a telehealth monitor in advance
- Digital pedometer FitBit Ultra registered the number of steps
- ActiveHeart is an interactive Web portal with information on heart functions, heart diseases and symptoms, videos with instructions on exercises, and brief rehabilitation narratives by patients and relatives. There was also a Web forum enabling patients to communicate with each other. The Web forum was moderated by a nurse. ActiveHeart.dk was developed by Aalborg University.
- Electronic rehabilitation plan (digital rehabilitation plan) giving an overview of patient data including goal and plan for rehabilitation, appointments, diary, and an overview of measured values (pulse, blood pressure, weight, and steps). Patients, relatives, and health care professionals had access to the plan. The Shared Care Platform was developed by IBM Corp.
- Android tablet (Samsung Galaxy Tab 2, 10.1) for accessing the interactive Web portal and digital rehabilitation plan.

Exercise:

- Individual and group-based aerobic and strength training

Education:

- Individual and group-based education within the following themes: self-management, physical activity, nutritional counseling, medications, psychosocial support, and managing a new lifestyle

### Theory

The theoretical framework for this case study consists of the “community of practice” approach [17] and SDT [18,19]. Wenger defined a “community of practice” as a group of people who share a concern or passion for something they do and who interact on a regular basis. This approach was applied to this study to determine whether the TTP can stimulate the emergence of communities of practice among patients, partners, and health care professionals.

SDT [24] focuses on human motivation, in general, and more specifically, whether a telerehabilitation program based on SDT can help motivate cardiac patients and their partners to improve their disease management skills and enhance their participation in rehabilitation. SDT describes how human beings inherently strive to develop or grow psychologically and how external factors may either impede or support this process. As such, SDT also provides a theoretical framework for understanding long-term motivation, as extrinsic motivation (ie, being prompted by others to perform a certain behavior) persists only as long as the external motivator is present. In contrast, intrinsic motivation refers to internalized motivation (ie, no external motivators are needed for the motivation to persist). To experience intrinsic motivation, it is necessary that all three basic human needs are sufficiently fulfilled: autonomy (acting in accordance with one’s internal values), competency (having the necessary knowledge and skills), and relatedness (being recognized by others) [18,19]. Motivation can be nurtured by supporting the patient’s experience of having these three basic needs sufficiently fulfilled simultaneously.

Key themes from the community of practice and SDT theories were applied in the observation and interview guides used in the data collection process (Multimedia Appendix 2, Multimedia Appendix 3).

### Data Collection Techniques

A triangulation of data collection techniques was used, such as documentary materials, participant observation, and qualitative interviews with patients and partners. Multiple sources of evidence were used to help provide multiple measures of the same phenomenon [20] in the case study. Data from documentary materials, participant observation, and interviews were analyzed in the same process, as the triangulation of data helped enhance the validity of the case study.

### Document Analysis

As background for the study, documents and reports on rehabilitation strategies, policies, and homepages from the involved health care organizations were reviewed. The aim of using documents was to achieve a basic understanding of the context of cardiac rehabilitation activities for the case study. Documents were analyzed using NVivo 11.0 (QSR International, Melbourne, Australia).

### Participant Observation

An observational guide (Multimedia Appendix 2) was developed based on the theoretical framework. The aim of performing participant observation [25] was to observe the interaction between patients and health care professionals in their telerehabilitation activities, patients’ participation in the TTP, and patients’ interaction with their partners using the technologies. Observations took place during meetings between patients and health care professionals on discharge from the hospital, when patients were introduced to the telerehabilitation program and technologies and at the commencement of rehabilitation carried out at the health care center. Observations also took place in patients’ homes, focusing on the patients’ use of the telerehabilitation technologies in everyday life and in their interaction with partners. The majority of the observations were carried out in the patients’ homes.

The observations were conducted by the first author (BD), who has a nursing and social science background, and the last author (HS), who has a background in Psychology. A total of 72 hours of participant observation was carried out. Notes were taken and documented in a Microsoft Word (Redmond, WA) file and analyzed using NVivo 11.0.

### Interviews with Patients and Partners

As per the study by Kvale and Brinkmann [26], semistructured qualitative interviews were conducted with cardiac patients and relatives, first at the time of enrollment into the TTP and then after 12 weeks of participation. The aim of the initial interviews was to gain an understanding of the patients’ disease and rehabilitation plan and the everyday life of the patient and his/her partner. The aim of interviews after 12 weeks of participation in the TTP was to explore the experiences of the cardiac patients and their partners.

Both patients and their partners were interviewed at the same time to ensure openness in the situation, although we were aware that it could be a barrier for some patients and their partners. One (single) patient wanted a son to represent a “partner’s point of view” due to their close daily relationship. In order to exclude topics that the patient and partners might not want to discuss together, the patient and partner were told prior to the interview that they could take a break in the interview to postpone talking about the topic until later when they could talk to the researcher on the phone, or to refuse talking about a topic. No patients or partners (nor the son) asked to delay talking about topics.

On enrollment, the main dimensions of the interviews were introduction; everyday life, limitations imposed by the disease on everyday life, family life, experience with use of technology, expectation to use of telerehabilitation technologies, social network, management of illness in everyday life, co-operation with partner, co-operation with health care professionals, and expectation for the future. After the patients had participated in the TTP for 12 weeks, the main dimensions in the interview guide for both the patient and their partner were influence of TTP on daily life, possibilities and limitation taking measurements, experiences with use of technologies (e-rehabilitation plan, step-counter, and ActiveHeart), social network (including other cardiac patients), managing one’s own rehabilitation supported by technology (information about rehabilitation, goal setting, and co-operation with partner and health care professionals), and experiences in participating in the TTP. A sample interview guide is provided in Multimedia Appendix 3.

The interviews were conducted by the first (BD) and last author (HS). The researchers had no relationship with the patients or their partners prior to commencement of the study. The interviews were tape recorded and transcribed by a research assistant. The interviews took place in the homes of the patients and lasted between 90 and 120 minutes. Interviews were conducted until we reached the point of data saturation.

### Analysis

All data from documents, notes from participant observations, and interviews were analyzed using NVivo 11.0. The first (BD) and last authors (HS) designed the code tree and performed the analysis. As per the study by Kvale and Brinkman [26], the analysis was carried out in the following steps:

1. Key themes and definitions from the theoretical framework were identified by the authors.
2. All data from documents, observations, and interviews were reviewed to obtain an overall impression of the themes on how the cardiac patients and their partners experienced participation in the TTP.
3. Based on key definitions and concepts derived from the theoretical framework and from reviewing observations and interviews, a code tree was designed (Textbox 2).
4. Before coding the data, the code tree was reviewed and discussed by the first (BD) and last authors (HS) to ensure intersubjectivity.
5. In the first step of the coding process, it was important to gain an understanding of the patient’s and his/her partner’s self-understanding.
6. In the next step, the interpretation was widened to include an understanding beyond the patient and their partner. What motivated their views and what was the scope of action?
7. In the final phase, the data were analyzed with a focus on the experiences of cardiac patients and their partners in participating in the TTP.
8. The data have been condensed and are presented in Textboxes 3 and 4 in the form of key themes and findings.

**Code tree.**

Use of technology in everyday life:

- Use of ActiveHeart Web portal
- Electronic rehabilitation plan

Participating in the Teledialog Telerehabilitation Program as a patient (autonomy):

- Feeling of self-control

Feeling of relatedness:

- Partner
- Family
- Health care professionals
- Other cardiac patients

Competencies:

- Technologies as a toolbox
- Empowerment in everyday life

Partner’s view:

- Use of ActiveHeart
- Rehabilitation activities and e-rehabilitation plan
- Being a partner

**Findings from cardiac patients participating in the Teledialog project.**

Use of Technology:

- ActiveHeart.dk was used as a “virtual book” on education.
- Electronic rehabilitation plan provided active assistance in defining and continuously reviewing goals and plans for rehabilitation activities and ensuring continuity in the rehabilitation process.

Autonomy:

- Flexibility and possibilities for individual decision making in the Teledialog Telerehabilitation Program.
- The Teledialog Telerehabilitation Program encouraged patients to carry out activities with their own initiative.

Relatedness:

- Health care professionals become coaches in aiding patients’ return to everyday life.
- Partners were considered important sources of support for continuing training.
- No feeling of being part of a community of practice with other cardiac patients.
- Web forum was not used because it became too private.

Competences:

- Telerehabilitation technologies and data overview encouraged patients to integrate rehabilitation into their everyday life.
- Patients were better able to perform rehabilitation activities outside hospitals and health care centers despite the need to plan time and geographical distance.

**Findings from the partners participating in the Teledialog project.**

Use of technologies:

- ActiveHeart.dk is a useful tool for the patient’s partner.
- E-rehabilitation plan is helpful in coordinating goals and creating overview of partner’s rehabilitation activities.

Being a partner:

- Mutual agreement on goals for planned rehabilitation activities facilitated coherence
- Sharing online information created a sense of security

- Partners took too much responsibility reviewing data on behalf of their partner
- Balance between being overly involved and being an equal partner
- Pushing the patient too “fast forward” in the rehabilitation process

### Ethical Approval

The project was approved by the Danish Ethical Committee (N-20120051) and performed according to the Declaration of Helsinki. All participants signed an informed consent agreement. The Teledialog Project is registered at ClinicalTrials.gov (NCT01752192). The study followed the guidelines in the Danish Act on Processing of Personal Data.

## Results

### Key Themes and Findings

In Textboxes 3 and 4, key themes and findings from patients and their partners are listed and described in more detail with quotations from patient and partner interviews.

We found no differences in perceptions based on sex, age, type of disease, or illness among the cardiac patients and their partners. As shown in Table 1 on baseline characteristics, the men and women among patients and their partners were within the same approximate age group.

### Use of Technology

Both patients and their partners expressed the view that the ActiveHeart.dk was a useful tool for patient education in the rehabilitation process and that it provided them with relevant information in text and video about heart diseases, symptoms, and topics on changing lifestyle:

I have been able to find relevant information in the ActiveHeart.dk on topics about rehabilitation. I have used the videos of how to do exercises very much, and the video with other cardiac patients describing how they were feeling has been helpful for me and my wife.

Patient #15, male

The e-plan has been the backbone of my rehabilitation process between me, the healthcare professionals and my wife.

Patient #19, male

A partner expressed her views about the ActiveHeart.dk:

It has been very helpful to have all information online and available 24/7, and especially the videos with narratives from partners have been very informative and made me feel secure.

Partner #16, female

The patients’ and partners’ views about the e-rehabilitation plan generally indicated that the plan had given them an overview of goals, plans, and appointments, creating a sense of coherence in the rehabilitation process:

It is a relief to have the digital record and be able to follow the goals and plans made for my husband and which encouraged him to stick to the plans.

Partner #18, female

Integrating patient education, communication with health care professionals, and the e-rehabilitation plan using a single access point, namely, the ActiveHeart.dk Web portal, provided patients and partners with the necessary knowledge and overview of the rehabilitation process, resulting in a much-valued sense of coherence in the rehabilitation program.

### Autonomy

Our data indicate that the TTP encouraged the patients’ autonomy because they felt more involved in making personal decisions related to their rehabilitation process.

The patients felt they had control over their own situation. One patient reported the following in the interview:

The Teledialog project has provided the framework for individualized rehabilitation at a distance and stimulated me to do activities that I felt were useful for me in my situation.

Patient #21, male

Being able to view my own data and be ‘the captain’ of my own rehabilitation is important to me.

Patient #23, female

### Relatedness

During their participation in the TTP, cardiac patients felt that the health care professionals took on a new role as coaches in aiding their return to everyday life. The coaching revolved around the mutually accessed data (steps, weight, blood pressure, and pulse):

I have experienced the healthcare professionals in a new role as coaches, and I think this is because they can see how physically active, I am, and in this way, encourage me to keep me focused on my rehabilitation.

Patient #22, male

The partners became an important support resource in everyday life for the patients, motivating them to keep up their training lifestyle changes:

My wife is an excellent supporter for me when I do not feel like taking my daily walk or going to the healthcare center for training.

Patient #27, male

I appreciate it when my husband keeps pushing and motivating me to keep training and following my goals for my rehabilitation.

Patient #29, female

As such, the TTP enabled both health care professionals and partners to provide important feedback to the patient, who, in turn, experienced enhanced relatedness.

Some patients felt that by participating in the TTP, they were lacking a sense of belonging to a patient community of practice. They missed the contact with other cardiac patients, where they could exchange views and experiences about their lifestyle changes. Observational notes showed that the patients who missed having more contact with other cardiac patients were first-time cardiac patients:

I enjoy the idea of telerehabilitation, but I would have liked more possibilities to meet with other cardiac patients in order to exchange experiences after admission to the hospital.

Patient #29, female

This suggests that the TTP was not able to build an online interactive forum in which patients could experience patient-to-patient relatedness. Only a few patients used the Web forum:

I do not like to communicate on a web forum. I think that my rehabilitation and questions related to this are a private matter.

Patient #2, male

### Competences

Patients felt that the daily data summary they received was a good source of information about their competency with regard to their required rehabilitation activities. The data summary encouraged patients to perform their rehabilitation activities more comprehensively and more frequently, at any time or place, and independent of geographical distance (at home, work, or a summer house):

I get motivated because I can see how I perform on my physical activity and then carry out the activities whenever it fits into my daily schedule and working life.

Patient #27, female

Based upon [my rehabilitation] data, I feel motivated to keep focus on progress towards my goals and plans for my rehabilitation.

Patient #16, male

### Being a Partner

The partners viewed the e-rehabilitation plan as helpful in facilitating an understanding and coherence in the rehabilitation process for both themselves and the patient, thus creating a feeling of security:

Being involved in the process of goal-setting for the rehabilitation of my husband gave me an understanding of the importance of changing lifestyle and the challenges that can arise.

Partner #17, female

Some partners took too much responsibility during their participation in the TTP. They found it difficult to navigate between the desire to be involved, to be over-protective of their spouse, and to remain an equal partner in the rehabilitation process. The result could be a situation where they pushed the patient too hard in the rehabilitation process. Observations showed that this tendency occurred mostly in female partners:

I think that in the beginning of my husband’s rehabilitation process, I was very eager to help understand the data and define activities; e.g., walking more steps or losing weight. I felt that my husband became passive and was not engaged...we had a talk about my role, and I realized that I had become too involved. I think there is a balance about being involved and being an equal partner...I have now realized that rehabilitation takes time.

Partner #20, female

I had a difficult time balancing between motivating my husband to get going on a new lifestyle and at the same time not taking over… it resulted in me pushing my partner too hard, and he became frustrated.

Partner #22, female

## Discussion

### Principal Results

A triangulation of data was used in the case study, and the aim was twofold: (1) to provide multiple measures of the same phenomenon and (2) to construct validity of the case study.

The aim of the case study was to explore the experiences of cardiac patients and their partners in participation in the TTP. Findings showed that they found the technology, such as the ActiveHeart.dk Web portal and the e-rehabilitation plan, to be helpful tools in educating, coordinating (goal-setting), and creating an overview of their rehabilitation data and ensuring continuity in the rehabilitation process. The patients felt that the TTP make them feel like individuals and gave them a sense of autonomy, an increased relatedness to health care professionals and partners, and a sense of competence as active players in their own rehabilitation process. Some patients missed being part of a community of practice with other cardiac patients, and these patients did not use the Web forum option on ActiveHeart. Patients’ partners found that the telerehabilitation program gave them a sense of security and helped them navigate between involvement in the rehabilitation, being an equal partner as the patient, and not pushing the patient too hard.

### Interpreting the Findings in the Context of the Research Literature

Patients and health care professionals often have competing priorities and interests in terms of the kind of information they feel is most important during treatment [27]. Our findings highlight the fact that both patients and their partners found ActiveHeart and the e-rehabilitation plan to be useful tools in the rehabilitation process. The health care professionals from the Teledialog project stated that the e-rehabilitation plan facilitated knowledge and information sharing between themselves, the patients, and their partners [28]. Furthermore, the patients became active collaborators in their rehabilitation process [28]. Jansson et al [29] reviewed the use of health plans formulated between health care professionals and patients with acute coronary syndrome. Similar to our findings, they reported that shared information and shared decision making facilitate partnership between patients and health care professionals. In addition, we identified one ongoing study on cardiac telerehabilitation that used a personalized patient-centered Web app [30]. In this study, however, it was unclear whether the patients and their cohabiting partners could share information with health care professionals across sectors in the rehabilitation process, as was done in the Teledialog project.

In the Teledialog project, the patients did not seem to have any technological challenges, perhaps due to the inclusion criteria in this study, where the selected patients had to have user-level competence in information and communication technologies. In a substudy, the patients expressed the view that the ActiveHeart Web portal was easy to access, user friendly, and written in an understandable language [31].

Factors that might be obstacles to adoption of telehealth or telerehabilitation can be requirements for technical competences, operation of equipment, threats to identity, and attitudes that digital services undermine in self-care and independence [32]. A recent study on cardiac rehabilitees’ experiences with technology concluded that they had different experiences and expectations and they expect the technology to be simple, flexible, and easy to use and learn [33]. Albert et al [34] highlighted the importance of matching technologies with patients’ personal learning and use needs [34].

In our study, the cardiac patients felt that the TTP supported their sense of autonomy, giving them the possibility to construct individually tailored activities, and they felt motivated to carry out their rehabilitation tasks on their own initiative. Feeling free to plan one’s own activities and move about as desired is generally associated with a greater feeling of autonomy. This is confirmed in another qualitative substudy in the Teledialog project that focused on patients’ use of a digital pedometer [35]. We have not identified other studies that support this finding. Future research needs to explore this perspective in depth over time.

The TTP increased the degree of relatedness between patients, health care professionals, and patients’ partners. Patients found that the health care professionals became coaches for them in integrating their rehabilitation activities into their everyday life. Similar results have been found in a Danish study on patients with chronic pulmonary diseases, where a community of practice developed between patients and health care professionals who also took on a role as coaches for the benefit of the patients in their rehabilitation [36].

Some patients felt that they missed being part of a community of practice with other cardiac patients. Indeed, some patients might prefer to participate in rehabilitation activities at a health care center, where they can have more face-to-face contact and can feel part of a vibrant community of fellow patients and staff. One study, for example, found that patients can be reluctant to use new telehealth services if they place a high value on the existing services [32].

A few patients stated that they did not use the ActiveHeart Web forum for communication between each other. They considered this kind of open forum communication to be too private for their taste. One possible factor in explaining patients’ hesitancy to use the Web forum might be the age of the enrolled patients. Older persons tend to be less accustomed to using social media to communicate with each other. The Web forum did not seem to too important to our patients. We discovered that patients make choices about what kind of digital media they want to use. In our study, the patients made use of the other digital solutions. Hence, they could refuse to use the Web forum but still use other digital media or solutions. Users apparently make informed choices. The findings that patients did not feel part of a community and avoided using the Web forum indicate that a telerehabilitation approach might not be suitable to all cardiac patients.

When designing telerehabilitation programs and technologies, it is important to stimulate interactive relationships among the involved parties. Recognizing someone’s efforts also acknowledges the relationship and its importance, thus fulfilling the need for relatedness [19]. In the TTP, both patients and partners had access to the same technologies (data in the e-rehabilitation plan and the ActiveHeart.dk Web portal), and these technologies that might have helped them to be better able to communicate and share more concerns, anxieties, and questions.

In the SDT, the competency factor denotes the need to feel competent when taking on a task or new behavior [19]. The patients in our study stated that by participating in the TTP, they were able to integrate rehabilitation activities into their everyday and working lives. Being able to integrate rehabilitation into everyday life also makes it easier to master the “work” of being a patient. When rehabilitation becomes an extra task that needs to be fitted into an already busy schedule, the motivation to keep it up may diminish. We know that working patients find it difficult to participate in rehabilitation because of scheduling, and sheer distance to a health center may be another factor affecting the level of adherence [3].

Results from a survey in The Teledialog project comparing the telerehabilitation and control group showed that both groups were equally motivated for lifestyle changes and self-care and that they experienced a similar level of quality of life [37].

The goal of any telerehabilitation program is to allow patients the temporal and logistical freedom to organize their own rehabilitation process, thus giving them a competency-based motivation. They can focus on their rehabilitation process after other tasks have been completed, and they do not need to consider the time and expense of traveling to a rehabilitation center. This flexibility may also decrease the patients’ feeling of a disparity between being a patient and reintegrating into daily life. If coping with lifestyle changes and disease management are to be successful, the rehabilitation activities need to become part of people’s everyday life; hence, telerehabilitation can facilitate this need for enhanced integration.

Our findings indicated that integrating a partner into a telerehabilitation process gave additional partner involvement and allowed partners to become more engaged than they could be in conventional rehabilitation. The additional involvement factor was attributed to the functionalities of the technologies used. Jansson et al [29] concluded that the involvement of patients, partners, and stakeholders led to person-centered care that included maintaining social relations and being able to maintain other activities, including regular work. Person-centered care or treatment trends are certainly going to become a more common health care strategy in the future [38].

Both patients and partners expressed a sense of security related to taking part in the telerehabilitation program. At the same time, patients’ partners acknowledged the challenges of navigating between being involved, being an equal partner, or not pushing the patient too hard. The enhanced sense of security felt by both patients and their partners was also a positive finding of our study, in view of the fact that overprotection is a common problem in couples dealing with cardiac disease [14]. Overprotective or controlling behavior by the cohabiting partner often diminishes the couple’s ability to cope communally with the challenges of rehabilitation [14]. An overprotective or controlling partner may reflect their anxiety about the patient falling ill again or worsening of the disease. The ability to release some of the understandable anxiety was related to a partner’s serious disease and having one’s questions answered, and concerns addressed by health care staff make it possible for partners to engage more actively in the relationship and to assist their partner in facing rehabilitation challenges. Hence, the couple relationship, instead of being a source of tension, may serve as a source of social support for both the patient and partner. Social support has been strongly associated with better outcomes. However, the degree of positive social support is dependent on the quality of the relationship [15]. We must also be aware that partners’ tendency toward controlling and overprotective behavior can be reduced by recognizing their important role in rehabilitation and by involving them in the rehabilitation process from the earliest stages.

### Limitations

Case studies that are properly carried out can provide grounds for generalization [39]. First, in order to ensure the validity of our case study, a triangulation of data collection techniques was used. Interviews with the patients and partners took place at the same time. It is certainly possible that the presence of patients together with their partners might have influenced the conversation and the degree of respondents’ openness during the interviews. However, we chose to conduct the interviews in pairs in order to have openness in the interview situation.

Second, we used a software program to analyze data. This kind of analysis may decontextualize the data on the rehabilitation sequence, leading to loss of valuable insights into what is certainly a complex process.

We are aware that the results and discussion on telerehabilitation on the motivation of cardiac patients do not allow us to fully discuss this aspect in this qualitative study. However, we have explored this issue in a survey in the Teledialog study [37].

### Conclusions

Cardiac patients and their partners found telerehabilitation technologies a helpful digital toolbox in the rehabilitation process. Telerehabilitation motivated the patients to integrate rehabilitation activities into their work schedule and everyday life and made them feel like unique individuals. Participating in a telerehabilitation program might not be a suitable strategy for all cardiac patients. Being a patient’s partner in a telerehabilitation program was associated with a heightened sense of security about the process, and the partners had to navigate between their involvement in the process, being an equal partner, and not pushing the patient too hard. Future research needs to focus on longitudinal case studies of both patients’ and their partners’ motivation in order to determine the benefits of participation in a telerehabilitation programs.

## Appendix

Multimedia Appendix 1 Teledialog video.

Multimedia Appendix 2 Observation guide.

Multimedia Appendix 3 Interview guide for patients and their partners.

## Abbreviations

N/A: not applicable
SDT: Self-determination Theory
TTP: Teledialog Telerehabilitation Program
