# Supplementary figures and images for: Integration of Rehabilitation Activities Into Everyday Life Through Telerehabilitation: Qualitative Study of Cardiac Patients and Their Partners
Source: J Med Internet Res. 2019 Apr 15;21(4):e13281. doi: 10.2196/13281 (PMC6487348; doi:10.2196/13281)

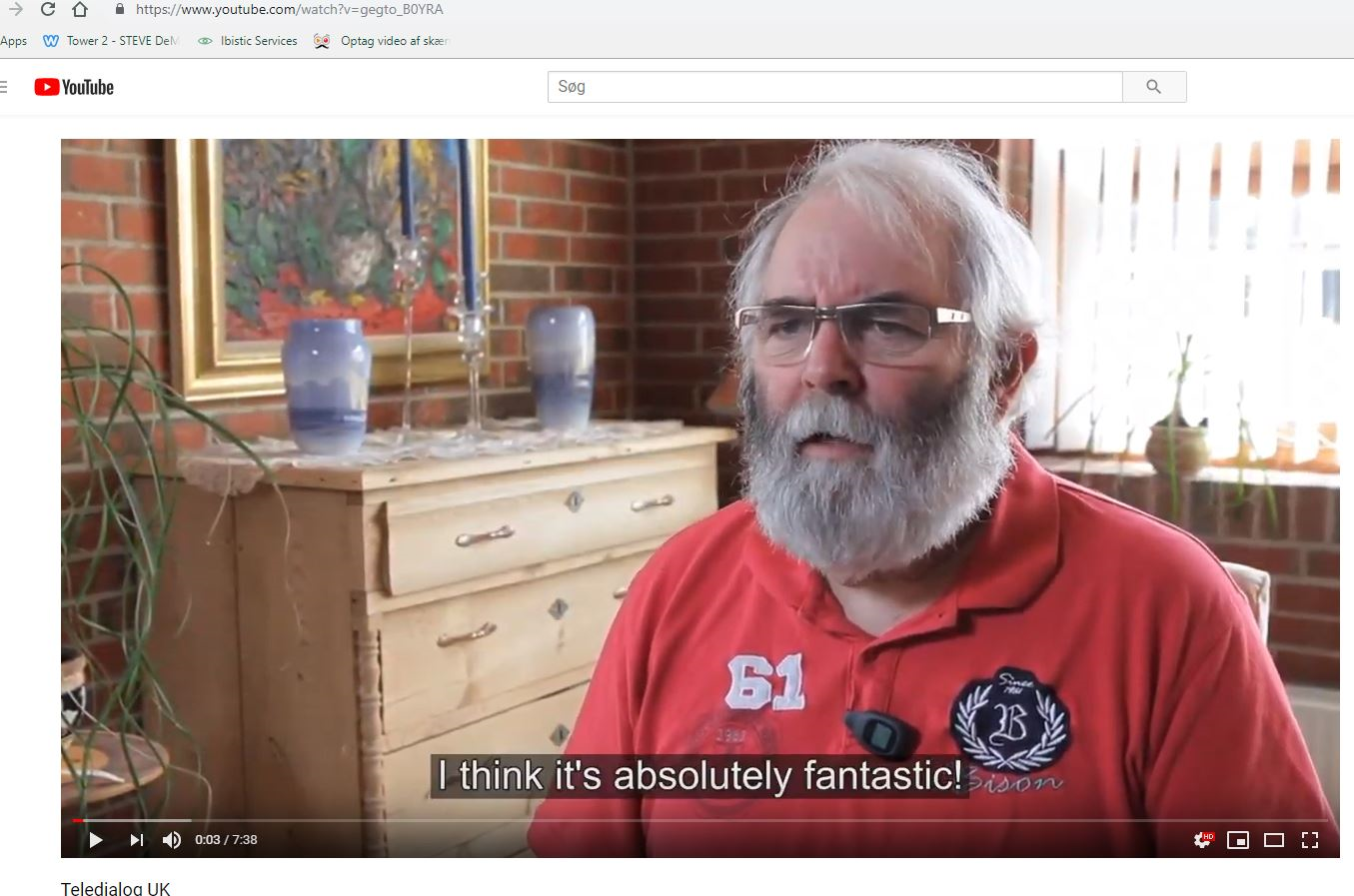

Supplement: Multimedia Appendix 1 [file jmir_v21i4e13281_app1.png]
