# Supplementary material for: Integration of Rehabilitation Activities Into Everyday Life Through Telerehabilitation: Qualitative Study of Cardiac Patients and Their Partners
Source: J Med Internet Res. 2019 Apr 15;21(4):e13281. doi: 10.2196/13281 (PMC6487348; doi:10.2196/13281)
Supplement: Multimedia Appendix 2 [file jmir_v21i4e13281_app2.docx]

# Appendix 2: Observation guide

**Themes for observations**

1. Interaction between patients and healthcare professionals

- Conversation on identification of the individual patients’ needs for rehabilitation activities
- Patients’ and partners’ introduction to telerehabilitation technologies

1. Patients’ participation in the TTP

- Activities at home (measuring values, searching at the ActiveHeart portal, following measured values over time during the TTP, communication with healthcare professionals)
- Activities at the healthcare center (conversations with nurse or physioperapist on the status, e-rehabilitation plan, training sessions with other cardiac patients)
- Rehabilitation, everyday life and work life

1. Patients’ interaction with partners
   - Conversation on rehabilitation issues
   - Everyday life and rehabilitation activities
