# Supplementary material for: Integration of Rehabilitation Activities Into Everyday Life Through Telerehabilitation: Qualitative Study of Cardiac Patients and Their Partners
Source: J Med Internet Res. 2019 Apr 15;21(4):e13281. doi: 10.2196/13281 (PMC6487348; doi:10.2196/13281)
Supplement: Multimedia Appendix 3 [file jmir_v21i4e13281_app3.docx]

# Appendix 3: Interview guides

Interviews with the cardiac patients and their partner took place at the same time. The `you´ in each question refers to both the patient and partner.

**Abbreviation:**

Teledialog Telerehabilitation Program (TTP)

| **At enrolment** | **12 weeks after enrolment** |
| --- | --- |
| **Presentation**   1. Would you like to introduce yourselves? | **Introduction**   1. How have things been going since the last time we spoke? |
| **Your everyday life**   1. What is your everyday life like?    - Rhythm of the day    - Work    - Leisure time    - Activities 2. Does your illness create any limitations for you in your everyday life?  - What symptoms to you have? - How did the symptoms come?  1. Can you tell me about your family life? 2. Can you tell me about your leisure time interests?    - Are there things you do together? | **Your everyday life**   1. What influence has the TTP had on your daily life (lives)? Possibilities/limitations?  - Rhythm of the day - Work - Activities - Family life - Leisure time interests  1. What do you think about having had the possibility to take your own measurements? Possibilities/limitations? |
| **Use of technology**   1. Do you use a computer? A cell phone? Tablet or other devices? 2. What expectations do you have to the following technologies?    - Monitor    - e-rehabilitation plan    - FitBit (digital step counter)    - ActiveHeart.dk 3. How do you expect that you will use the technologies? | **Use of technology**   1. How have you experienced the use of the following technologies?    - Monitor    - e-rehabilitation plan    - FitBit (digital step counter)    - ActiveHeart.dk      1. What have you used the technologies for? In which situations? 2. Which opportunities have the technologies given you in terms of rehabilitation activities? Which limitations? |
| **Social network**   1. Do you have contact with other heart patients and relatives? Why/Why not? In this area, what would you want? 2. How do you maintain contact with your network beyond the family? | **Social network**   1. Have any changes occurred in your contact with others? Have you developed a new network? |
| **Managing your illness**   1. How do you manage your illness in your daily life?  - Exercise in your daily life/training - Administration of medication - Diet - Rest - Do you receive help from your relatives? - Do you search for information about your illness on the internet/other places?  1. How do you experience cooperation with your partner, the staff at the hospital, health center, general practitioner, etc.?  - How do you think the staff understands your everyday needs? - Does the healthcare staff motivate you to try out new ideas in your management of your disease (’help for self-help’)?  1. Is there anything you would like to do differently in your situation?  - Cooperation with the healthcare staff? - Communication with the staff about your situation? - Activities? | **Managing your illness**   1. What significance has it had for you and how you manage your disease:  - That you yourself could measure your own values (blood pressure, pulse, weight and steps)? - Has it had any physical impact? - Has it had any impact in relation to your training effort? - Have your partner helped you? How have you experienced this? - Do you do anything differently in relation to how you manage your disease?  1. What has it meant for both of you that you have had your own e-rehabilitation plan?      - Knowledge about disease? - Goalsetting and action plan for rehabilitation activities? - What have you learned? And how? - Has the use of the e-rehabilitation plan given you any ideas? - What has it meant to you?  1. How have you experienced to participate in the TTP? 2. Is there anything you would consider doing differently in your situation?  - Cooperation and communication with your partner? - Cooperation and communication with healthcare staff? |
